# **NU-9** improves health of hSOD1^G93A^ mouse upper motor neurons in vitro, especially in combination with riluzole or edaravone

**DOI:** 10.1038/s41598-022-09332-4

**Published:** 2022-03-30

**Authors:** Barış Genç, Mukesh Gautam, Benjamin R. Helmold, Nuran Koçak, Aksu Günay, Gashaw M. Goshu, Richard B. Silverman, P. Hande Ozdinler

**Affiliations:** 1 Department of Neurology, Feinberg School of Medicine, Northwestern University, 303 E. Chicago Ave, Chicago, IL 60611 USA; 2 Department of Chemistry, Northwestern University, Evanston, IL 60208 USA; 3 Department of Molecular Biosciences, Chemistry of Life Processes Institute, Center for Developmental Therapeutics, Northwestern University, Evanston, IL 60208 USA; 4 Department of Pharmacology, Feinberg School of Medicine, Northwestern University, Chicago, IL 60611 USA

**Keywords:** Phenotypic screening, Motor cortex, Motor neuron disease, Neurodegenerative diseases

## Abstract

Even though amyotrophic lateral sclerosis (ALS) is a disease of the upper and lower motor neurons, to date none of the compounds in clinical trials have been tested for improving the health of diseased upper motor neurons (UMNs). There is an urgent need to develop preclinical assays that include UMN health as a readout. Since ALS is a complex disease, combinatorial treatment strategies will be required to address the mechanisms perturbed in patients. Here, we describe a novel in vitro platform that takes advantage of an UMN reporter line in which UMNs are genetically labeled with fluorescence and have misfolded SOD1 toxicity. We report that **NU-9**, an analog of the cyclohexane-1,3-dione family of compounds, improves the health of UMNs with misfolded SOD1 toxicity more effectively than riluzole or edaravone, -the only two FDA-approved ALS drugs to date-. Interestingly, when **NU-9** is applied in combination with riluzole or edaravone, there is an additive effect on UMN health, as they extend longer axons and display enhanced branching and arborization, two important characteristics of healthy UMNs in vitro.

## Introduction

There is an urgent need to improve the success rate of clinical trials, and that depends on our ability to obtain knowledge that translates to patients. Comparing mice survival to human survival has not been translational. However, UMNs in mice and UMNs in humans share many common features of motor neuron biology and display identical characteristics of neuropathology at a cellular level^1–5^. Therefore, information obtained directly from UMNs of well-defined mouse models of motor neuron disease should be faithfully recapitulated in the UMNs of patients at a cellular level. Shifting our focus from mice to affected neurons appears to be a path forward to identify compounds that will improve the health of neurons that degenerate in patients^6–8^, so that the circuitries they are involved in will improve with functional outcomes. Furthermore, drug companies and the FDA now demand more information on the efficacy of compounds at a cellular level, as they also appreciate the importance of developing therapies that target the core of the problem.

To date, the FDA has approved only two drugs as being effective in ALS; the first is riluzole and the more recent one is edaravone^9^. Both drugs are effective for two distinct cellular events. Riluzole is thought to reduce glutamate-mediated excitotoxicity^10–12^, primarily at the presynaptic level, blocking the release of glutamate^13^. Proposed mechanisms of action of riluzole include the inhibition of voltage-gated sodium channels, noncompetitive inhibition of NMDA receptors, inhibition of glutamate release, and enhanced astrocytic uptake of extracellular glutamate^13^, but its precise target and mechanism of action requires further investigation. Edaravone, on the other hand, works as a free radical scavenger and was previously prescribed for stroke patients^14–19^.

Riluzole was approved for the treatment of patients with ALS in 1995, based on the results of two clinical trials where it had a significant effect on the rate of survival^20,21^. Clinical trials were initiated before riluzole could be tested on hSOD1^G93^^A^ mice^22,23^. Despite initial successful clinical reports, later studies in mouse models of hSOD1^G93A^,^24–29^ FUS^25^, or TDP-43^30^ failed to show significant improvement of life-span in mice with riluzole treatment, further suggesting lack of translation between extended survival in mice and in patients. Riluzole was also never tested for its ability to improve the health of diseased UMNs.

Edaravone, approved in 2017, was tested in hSOD1^G93A^ mice prior to its approval where it significantly slowed motor decline, reduced oxidative stress, motor neuron loss, and mSOD1 deposition in the spinal cord^15,31^. It is reported to act mostly on free radicals and detailed information on cellular events that contribute to improved motor neuron survival are still emerging^14,32^. However, there had been no report on its ability to improve the health of diseased UMNs in ALS.

**NU-9** was initially identified based on its ability to reduce misfolded SOD1 aggregation in cell lines, it crosses the blood brain barrier, has favorable drug-like properties^33–37^. When hSOD1^G93A^ and prpTDP-43^A315T^ ALS mouse models were treated with **NU-9** via daily gavage (100 mg/kg/day), UMN degeneration was reduced and structural integrity of mitochondria and ER were improved in diseased UMNs^38^. Importantly, **NU-9** treated mice also showed improved motor function behavior^38^, as assayed by inverted mesh assay^39^.

The motor neuron circuitry fails in ALS as the UMNs and the spinal motor neurons first become vulnerable and then begin to degenerate. An effective and long-term treatment would include strategies that improve the health and stability of diseased motor neurons, both in the brain and in the spinal cord, so that the motor neuron circuitry can remain functional in patients.

Currently there are no preclinical screening or validation platforms that incorporate or investigate the health of diseased UMNs. In an effort to include UMNs into preclinical drug discovery studies, we recently generated a reporter line for UMNs, UCHL1-eGFP mice, in which UMNs are genetically labeled with eGFP expression that is stable and long-lasting^40^. By crossbreeding with disease models that display UMN vulnerability and progressive degeneration, such as hSOD1^G93^^A^ mice^41^, we generated UMN reporter lines of disease models. Most importantly, because UMNs express eGFP, they can be distinguished among thousands of other cortical cells and neurons , and their cellular responses to compound treatment can be quantitatively assessed at a cellular level with precision that is not otherwise possible^4,7,8,40^. This represents a paradigm shift in drug discovery efforts by performing comparisons at the cellular level and utilizing the response of diseased UMNs to compound treatment as a direct readout.

Here, in this study, both riluzole and edavarone were investigated together with **NU-9**, especially for their ability to improve axon outgrowth, branching, and arborization of diseased UMNs in culture. Both extended axon outgrowth^42–46^ and increased branching and arborization^46–49^ have been trusted parameters that are used to assess improved neuron health by many different groups and for many different neuron types. We find that healthy UMNs have longer axons and have increased branching/arborization and this can be used to investigate their cellular responses to treatment.

We report that **NU-9**, the first compound reported to improve neuronal integrity of UMNs that are diseased due to mSOD1 toxicity and TDP-43 pathology *in vivo*^38^ was more effective than riluzole or edaravone in enhancing UMN axon outgrowth as well as branching and arborization in vitro. Even more significantly, diseased UMNs displayed a much improved response when **NU-9** was applied in combination with riluzole or edaravone. This additive effect of compounds that act by different mechanisms is rather important for building a mechanism-focused drug discovery effort for complex diseases, such as ALS.

## Results

### Identification of **NU-9** as the candidate

A 7-day repeat dose toxicity study was performed at the Laboratory Animal House (LAH), Toxicology studies were performed by Sai Life Science Ltd. (Chrysalis Enclave, Phase II, Hinjewadi, Pune—411 057, Maharashtra, India). No mortality was observed when **NU-9** was administered to male BALB/c mice once daily for seven consecutive days by oral gavage at a 100 mg/kg/day dosage. Following repeated oral dose administration of **NU-9** for seven consecutive days in male BALB/c mice, the plasma concentrations of **NU-9** on day 7 were quantifiable to 24 h with a T_max_ of 0.5 h. Brain concentrations on day 7 were quantifiable up to 24 h. When administered to mice at a dosage of 100 mg/kg/day via daily gavage, the concentration of **NU-9** detected in the CNS was 400 nM. 100 mg/kg/day of **NU-9** via daily gavage was determined to be the most effective dose *in vivo*^38^; therefore, a 400 nM dose was selected for the in vitro experiments.

### Axon length and Sholl plots are informative outcome measures for UMN health

**Figure 1:**
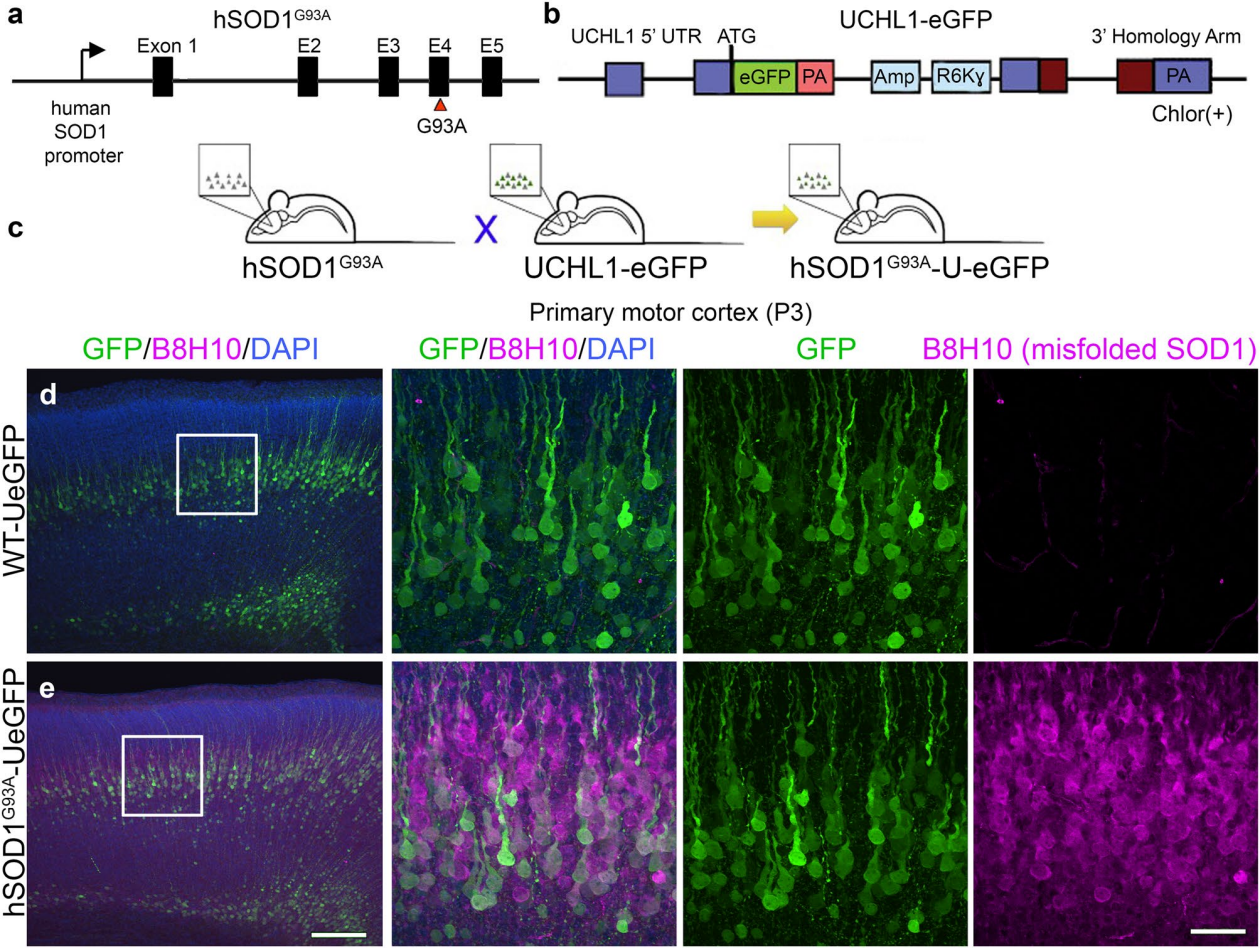
When mixed cortical cultures are established at P3, UMNs are labeled by eGFP expression in UCHL1-eGFP reporter mice, and express disease-causing proteins in reporter lines of mouse models of amyotrophic lateral sclerosis (ALS) (**a-c**) Generation of hSOD1^G93A^-UeGFP and WT-UeGFP littermate control mice. (**d-e**) UMNs in layer 5 of the primary motor cortex express eGFP at postnatal day P3 of WT-UeGFP mice. (**e**) B8H10 antibody detects misfolded SOD1 protein in the primary motor cortex of hSOD1^G93A^-UeGFP mice, but not the control WT-UeGFP mice at P3. Scale bars = 200 µm in low mag, 50 µm in high mag panels.

Recently, we generated and characterized a reporter line for UMNs. The UCHL1-eGFP reporter line expresses enhanced green fluorescent protein (eGFP) under the control of the UCHL1 promoter and genetically labels UMNs with eGFP expression that is stable and long-lasting, allowing visualization and cellular assessment of UMNs *in vivo*^40^ and *in vitro*^8^. Interestingly, crossbreeding of this reporter line with well-defined transgenic ALS mouse models did not alter their disease pathology, yet continued to allow visualization of UMNs that become diseased as a result of various underlying genetic causes (Fig. 1). For example, double transgenic hSOD1^G93A^-UeGFP mice were generated by crossing the UCHL1-eGFP with hSOD1^G93^^A^ mice (Fig. 1a-c), and they recapitulated the timing and extent of previously reported UMN degeneration^40^. For clarity purposes, we will refer to UCHL1-eGFP as WT-UeGFP mice, because it is the healthy WT control for the diseased hSOD1^G93A^-UeGFP mice.

Being able to visualize healthy and diseased UMN in culture among other cells and neurons has been exceptionally important, especially for drug discovery efforts, because it is now possible to study the impact of a candidate compound on UMNs with distinct pathologies. At P3, UMNs are already labeled by GFP expression in UCHL1-eGFP reporter mice (Fig. 1d-e)^40^. We have previously shown that disease causing misfolded SOD1, which can be detected by the monoclonal antibody B8H10, is present in the UMNs of hSOD1^G93^^A^-UeGFP mice^38,50^, and it can be detected as early as P3 in vivo (Fig. 1e). There is no B8H10 staining in the UMNs of WT-UeGFP mice (Fig. 1d).

**Figure 2:**
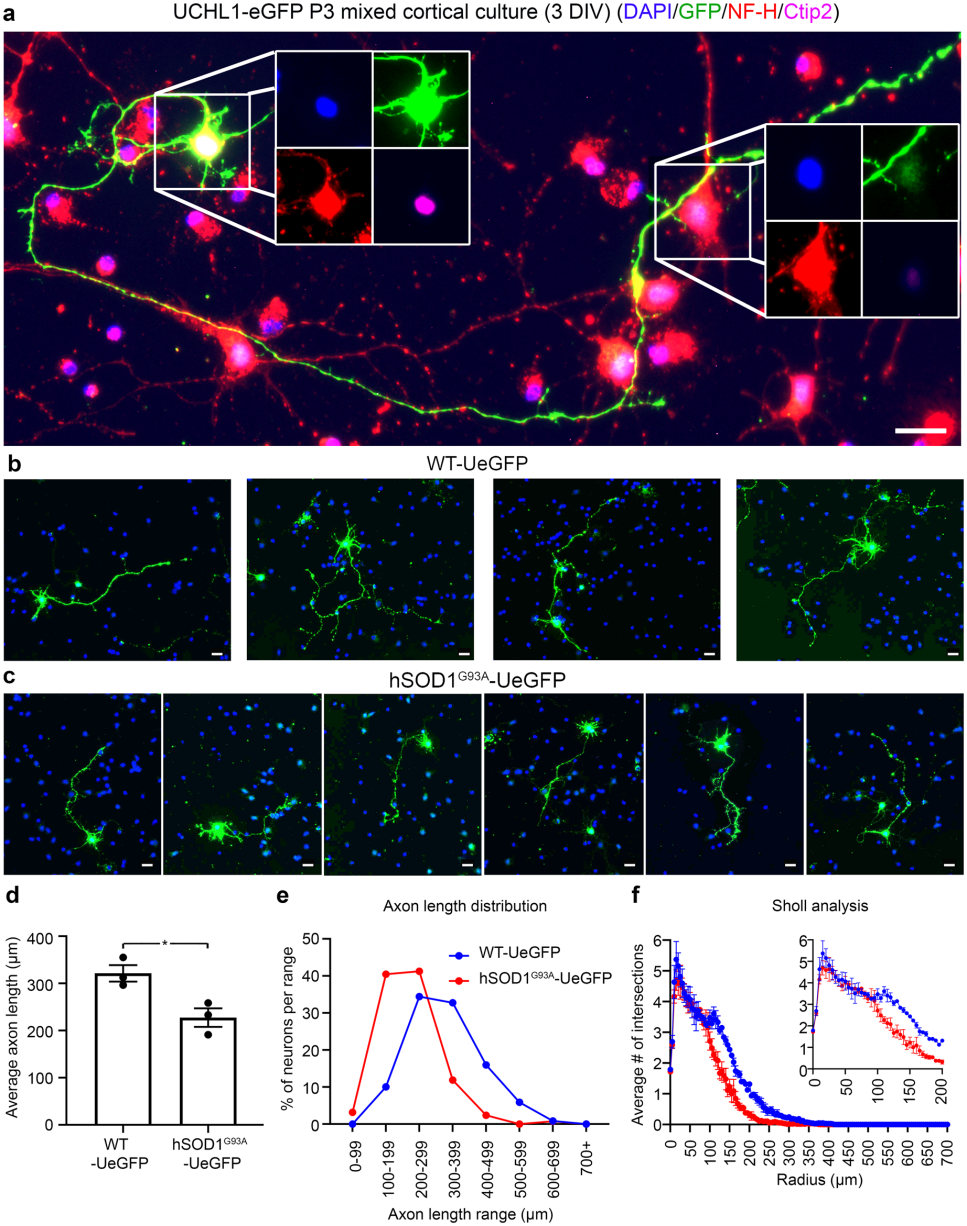
UMNs are distinguished from other neurons in culture, and changes in average axon length and branching/arborization offer quantitative outcome measures to investigate UMN health (**a**) UMNs retain their neuronal identity (NF-H + and Ctip2 +) and GFP expression in culture, and they can be distinguished from other neurons that are not an UMN (NF-H + , Ctip2 -). UMNs have large pyramidal cell bodies and long axons. (**b-c**) UMNs can be visualized and assessed individually in both control WT-UeGFP mice (**b**), and the hSOD1^G93A^-UeGFP ALS mouse model (**c**). (**d-e**) Bar graph representation of average axon length and (**d**) and percent distribution of UMN based on average axon length in WT-UeGFP (blue) and hSOD1^G93A^-UeGFP (red) mice. (**f**) Sholl analysis of healthy and diseased UMNs with 5 µm circular increments. 0–200 µm radius range is enlarged in the inset for clarification. Mean, SEM, and individual data points shown for *n* = 3 biological replicates. *p < 0.05, unpaired t-test with Welch's correction. Scale bars = 20 μm, n = 3 biological replicates.

When dissociated cortical cultures are established at postnatal day P3^46^ from WT-eGFP mice, the motor cortex is carefully dissected out from the rest of the brain to increase the percentage of UMNs in culture and to reduce the chance of introducing other eGFP + cells in the brain. UMNs retain their neuronal identity with large pyramidal soma, long axon, and they continue to express neuronal markers such as NF-H and UMN markers, such as Ctip2^8,51,52^ (Fig. 2a). In addition, the extent of their cell body can be fully visualized with eGFP expression, allowing detailed cellular assessments. Among many other cells (the blue dots (DAPI) represent nuclei of other cells) and neurons (as marked by NF-H) in culture, only the Ctip2 + , large pyramidal neurons express eGFP (Fig. 2a, Supplementary Fig. S1). The UMN identity of these neurons in the motor cortex were previously established by retrograde labeling, molecular marker expression and electrophysiology^8^. Therefore, we are confident that the eGFP + neurons in culture are indeed UMNs.

UMNs of WT-UeGFP mice (Fig. 2b) extend axons with an average length of 321.41 ± 17.54 μm (n = 119 neurons from n = 3 mice), whereas diseased UMNs of hSOD1^G93^^A^-UeGFP mice (Fig. 2c) extend a significantly shorter axon of 227.62 ± 19.63 μm (n = 122 neurons from n = 3 mice) vs. WT-UeGFP *p*-value = 0.024 (Fig. 2d, Supplementary Table S1). Investigation of dendritic branching/arborization of WT-UeGFP and hSOD1^G93A^-UeGFP UMNs using Sholl analysis revealed a significant difference between 100 and 225 µm radius distance from soma, meaning not only the axon length but also the branching patterns can be used as a measure of UMN health. The average axon length (Fig. 2d-e) and Sholl analysis (Fig. 2f, Supplementary Table S2) for WT-UeGFP and hSOD1^G93A^-UeGFP mice are significantly different by these two measures. Because average length of axon and the extent of neuronal branching/arborization serve as a quantitative outcome measure to distinguish healthy and diseased UMNs, these two independent parameters are used to assess their cellular response and overall health with respect to compound treatment.

### **NU-9** enhances axon outgrowth of diseased UMNs

**Figure 3:**
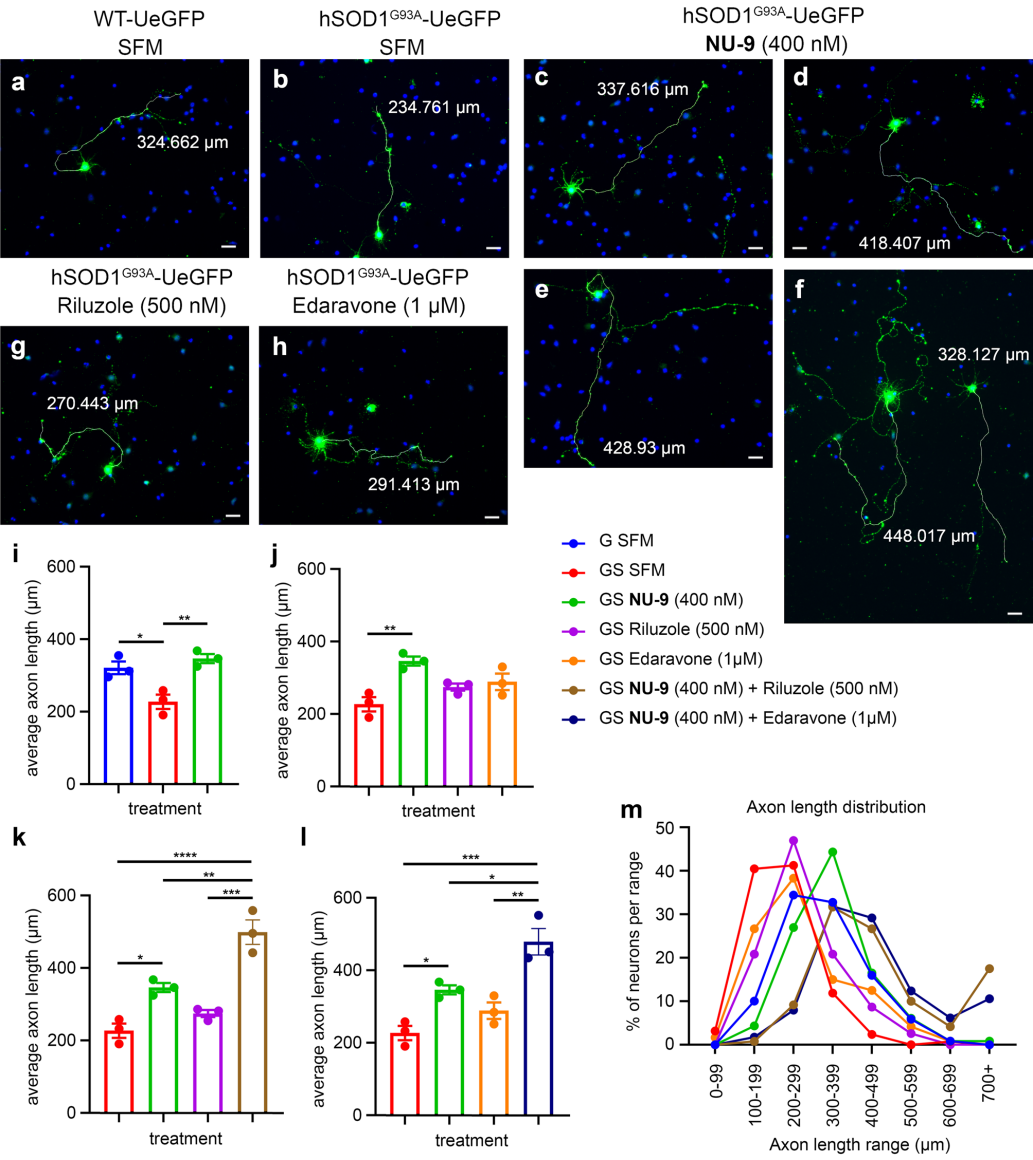
**NU-9** enhances axon outgrowth of UMNs that become diseased by mSOD1 toxicity. (**a**) Representative images of UMNs in dissociated cell cultures of motor cortex isolated from WT-UeGFP and (**b**) hSOD1^G93A^-UeGFP mice treated with SFM, (**c**-**f**) with 400 nM of **NU-9,** (**g**) 500 nM riluzole, (**h**) 1 µM edaravone for 3 days in vitro. Blue dots (DAPI) represent nuclei of other cells in culture, whereas UMNs are identified by their eGFP expression. (**i-l**) Average length of UMN axons in WT-UeGFP or hSOD1^G93A^-UeGFP mice treated with 400 nM **NU-9 (i)**, 500 nM riluzole, 1 µM edaravone (**j**), or combination of **NU-9** with riluzole (**k**) or edaravone (**l**). Mean, SEM, and individual data points shown for n = 3 biological replicates. *p < 0.05, **p < 0.01, *** *p* < 0.001, ****p < 0.0001, one-way ANOVA followed by Tukey's post hoc multiple-comparison test. (**m**) Percent distribution of UMNs based on axon length in WT-UeGFP or hSOD1^G93A^-UeGFP mice treated with 400 nM **NU-9**, 500 nM riluzole, 1 µM edaravone, or a combination of **NU-9** with riluzole or edaravone. Untreated WT-UeGFP (blue), untreated hSOD1^G93A^-UeGFP (red), hSOD1^G93A^-UeGFP treated with **NU-9** (green), edaravone (orange), riluzole (purple), NU-9 and edaravone (dark blue), **NU-9** and riluzole (brown). SFM = serum free medium; G = WT-UeGFP; GS = hSOD1^G93A^-UeGFP. Scale bars, 25 μm; n= 3 biological replicates.

Since axonal degeneration is an important contributor to UMN loss, any effective treatment strategy will require enhancement of the health and stability of UMN axons. We thus investigated whether **NU-9** was capable of promoting and enhancing axon outgrowth of diseased UMNs. Because UMNs are eGFP^+^ in control WT-UeGFP (Fig. 3a) and diseased hSOD1^G93A^-UeGFP (Fig. 3b) mice, they can be distinguished among other cortical cells and neurons in vitro, and their neuronal response to compound treatment is assessed at a cellular level with precision. To determine whether **NU-9** improves UMN axon outgrowth and whether this is comparable to that of previously approved drugs for ALS (i.e., riluzole and edaravone), we first introduced **NU-9** and drugs individually to the culture medium. To perform comparative analyses, the ALS drug concentrations that were previously calculated and reported to be present in the CNS after administration of the optimum dose used in clinical trials for riluzole (500 nM^10,21,24^), and edaravone (1 μM^15^) were used. Based on pharmacokinetic studies performed on **NU-9**, the dose detected in the CNS was 400 nM when administered to mice at a dosage of 100 mg/kg/day via daily gavage. Therefore, rather than assaying similar doses of compounds, the previously determined optimum active doses of **NU-9** (400 nM), riluzole (500 nM), and edaravone (1 μM) in the CNS were compared.

Diseased UMNs of hSOD1^G93A^-UeGFP mice extend a significantly shorter axon compared to UMN of control WT-UeGFP (227.62 ± 19.63 μm; adjusted p-value = 0.0177, Fig. 3i, Supplemental Table S1). Addition of **NU-9** to the culture medium significantly improved the average length of the UMN axons (346.76 ± 12.32 μm, n = 115 neurons from n = 3 mice; adjusted *p*-value = 0.0058; Fig. 3c-f,i), comparable to that of WT-UeGFP (adjusted *p*-value = 0.5657; Fig. 3i). Both riluzole (500 nM), and edaravone (1 μM) also slightly increased the average length of diseased UMNs, albeit they failed to reach significance (riluzole (500 nM): 274.83 ± 9.83 μm, n = 116 neurons from n = 3 mice; adjusted *p*-value = 0.2749, Fig. 3g,j; edaravone (1 μM): 289.58 ± 22.71 μm; n = 120 neurons from n = 3 mice; adjusted *p*-value = 0.1196, Fig. 3h,j). However, when **NU-9** was added in combination with riluzole or edaravone, the average length of the longest axon was longer than either drug alone (**NU-9** + riluzole: 499.29 ± 33.73 μm; n = 120 neurons from n = 3 mice; adjusted *p*-value compared to SFM < 0.0001, adjusted *p*-value compared to **NU-9** alone = 0.004, adjusted *p*-value compared to riluzole alone = 0.0003, Fig. 3k; **NU-9** + edaravone: 479.75 ± 36.72 μm; n = 113 neurons from n = 3 mice; adjusted *p*-value compared to SFM = 0.0004, adjusted *p*-value compared to **NU-9** alone = 0.0207, adjusted *p*-value compared to edaravone alone = 0.0026, Fig. 3l; Supplementary Table S1).

Plotting the distribution of the longest axons also revealed that the UMNs of hSOD1^G93A^-UeGFP mice cultured in SFM mostly had shorter axons (39.3% within the range of 100–199 μm and 41.8% within the range of 200–299 μm), whereas **NU-9** treatment caused an overall shift, resulting in about 44.4% of UMNs having an axon length in the range of 300–399 μm. Both riluzole and edaravone improved overall axon outgrowth (Fig. 3m), but not as profoundly as **NU-9**.

**Figure 4:**
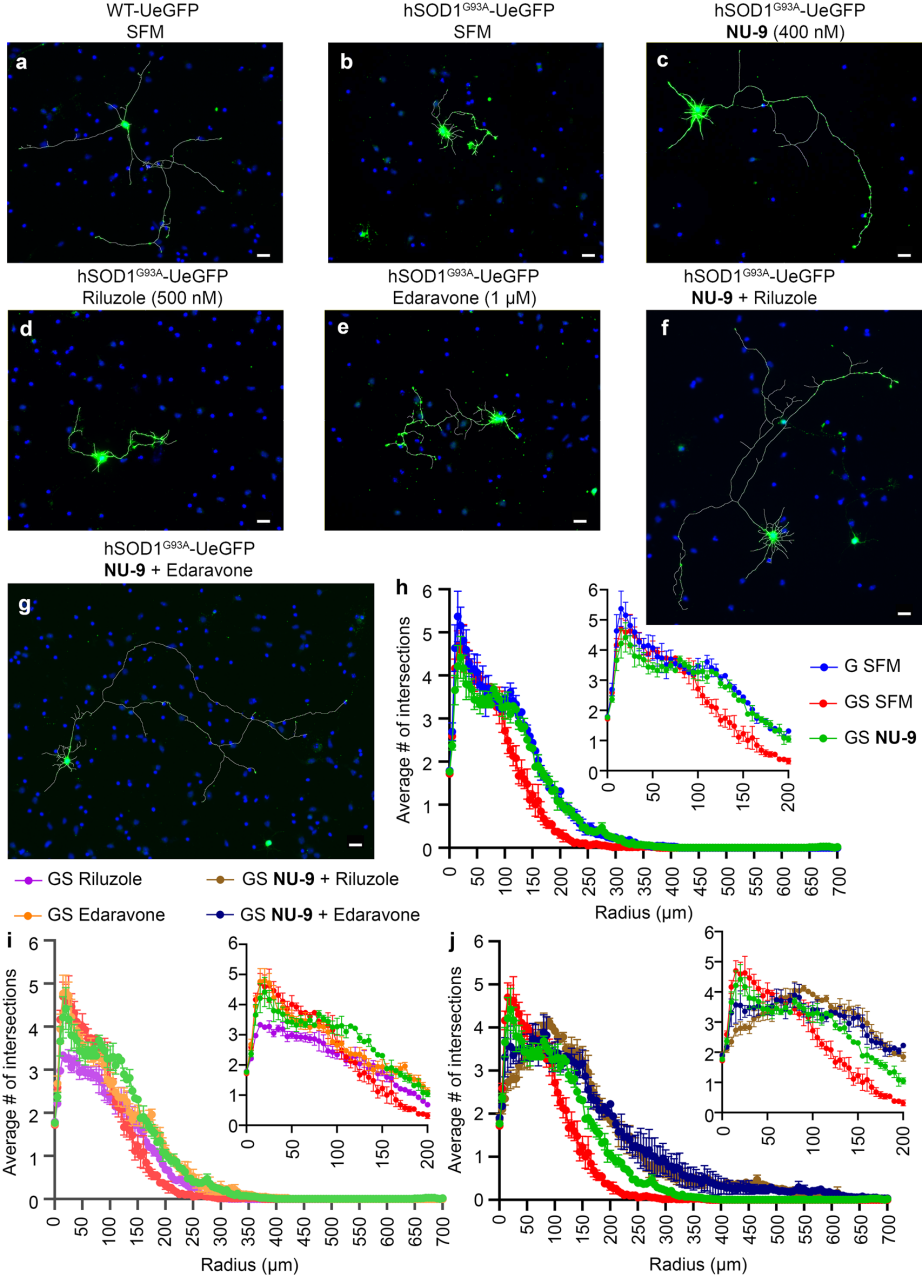
Sholl analysis of UMNs from WT-UeGFP and hSOD1^G93A^-UeGFP mice with or without various drug treatments. (**a**) Representative images of UMNs in dissociated cell cultures of motor cortex isolated from WT-UeGFP, hSOD1^G93A^-UeGFP mice treated with SFM (**b**), with 400 nM of **NU-9** (**c**), with 500 nM riluzole, (**d**), 1 µM edaravone (**e**), combination of **NU-9** and riluzole (**f**), combination of **NU-9** and edaravone (**g**) for 3 days in vitro. (**h**) Sholl analyses of WT-UeGFP UMN treated with SFM (blue), hSOD1^G93A^-UeGFP UMN treated with SFM (red), and **NU-9** (green). (**i**) Sholl analyses of hSOD1^G93A^-UeGFP UMN treated with **NU-9** (green), edaravone (orange), riluzole (purple), and SFM (red). (**j**) Sholl analyses of hSOD1^G93A^-UeGFP UMN treated with **NU-9** and edaravone (dark blue), **NU-9** and riluzole (brown), **NU-9** alone (green), and SFM (red). 0–200 µm radius range is enlarged in the inset for clarification. Scale bar = 20 µm, n = 3 biological replicates. SFM = serum free medium; G = WT-UeGFP; GS = hSOD1^G93A^-UeGFP.

Neuronal arborization and branching is yet another measure used to determine whether the health of the neuron is improved by compound treatment^53^. Both riluzole and edaravone also improved UMN arborization, but to a lesser extent (Fig. 4). Sholl analysis further confirmed that **NU-9** treatment resulted in the generation of more complex and arborized UMNs, even after 3 days in culture (Fig. 4, Supplementary Table S2). In the presence of **NU-9**, diseased UMNs not only extended longer axons, but they also displayed increased branching and arborization, a characteristic that is important for UMNs to recognize and innervate their targets (Fig. 4h). When **NU-9** was administered with riluzole (Fig. 4f) or edaravone (Fig. 4g), the average number of branch points significantly increased (Fig. 4i-j; Supplementary Table S2) and branching and arborization of diseased UMNs were enhanced when **NU-9** was applied together with riluzole or edaravone (Fig. 4j).

## Discussion

Neurodegenerative diseases were initially identified and characterized by clinical observations performed about a century ago. ALS was first characterized based on the progressive motor function defects detected in patients and with degeneration of the lateral column in the spinal cord, pointing to UMN loss and perturbations of cortical input to spinal cord targets^54^. Therefore, the importance of UMN degeneration, both for the identification and assessment of ALS pathology was essential, and it was required for proper diagnosis. However, over time the importance of UMNs was overlooked by the fact that patients die because of complications caused by muscles innervated by spinal motor neurons^55^. The idea that there is a linear degeneration that originates from the periphery and moves towards the brain, as a die-back phenomena, further diminished the need to investigate the survival requirements of UMNs with respect to ALS pathology^56,57^. Therefore, to date there had been no preclinical drug discovery platform that investigates the survival requirements of diseased UMNs.

When diseases were first clinically identified and characterized at the start of the century, the advances in science and technology were not sufficient to reveal the complexities of the diseases and to demonstrate that very many different underlying causes give rise to the same clinical outcome. Therefore, up until recently, the expectation was to find a drug or a treatment that is going to eradicate “the disease”. This expectation is now changing as FDA is also becoming more aware of the heterogeneity of the disease and that multiple treatment strategies may be needed to help patients^58^.

In recent years the underlying causes of ALS have begun to emerge, revealing the complexity and the heterogeneity of the disease. Therefore, the idea of developing mechanism-based treatment strategies gained momentum. In the novel approach described here, it became important to reveal the underlying causes of the disease in each patient and develop treatment strategies that would be effective for mechanisms that are perturbed in patients. This not only opens the door for “personalized medicine” approaches, but also demands a more and better understanding of combinatorial drug treatments, which have not been considered, especially for diseased UMNs^59^.

Since our goal is to develop long-term and effective treatment strategies for ALS and related motor neuron diseases, we investigated whether **NU-9**, the first compound reported to improve the health of diseased UMNs, was as effective as the previously FDA-approved ALS drugs, riluzole and edaravone. We also investigated whether **NU-9** treatment together with riluzole or edaravone would have an additive effect on the health of UMNs that are diseased due to mSOD1 toxicity.

In vitro cell culture assays offer immense potential to reveal cellular responses to treatment, especially if the neurons of interest can be distinguished among many different cortical cells and neurons in culture. We initially developed and characterized UCHL1-eGFP mice, in which the UMNs located in layer 5 of the motor cortex express eGFP. UMN identity of eGFP + neurons were confirmed by retrograde labeling, molecular marker expression, and electrophysiological properties^40^. Crossing this reporter line with well characterized ALS mouse models, such as hSOD1^G93^^A^ mice, helped generate reporter lines of UMNs. The advantage of these UMN disease models is that UMNs retain their eGFP expression both in vivo and in vitro, allowing their identification, visualization, and cellular assessment among many different cells and neurons. This is especially important for developing drug discovery or verification platforms in vitro, so that cellular responses to treatment can be monitored with cellular precision that was not possible before. Here we utilized dissociated cortical cultures established from hSOD1^G93A^-UeGFP mice, in which the UMNs are distinguished among other cortical cells and neurons by their eGFP expression, and they retain their UMN morphology and identity, as assayed by the expression of proper cellular markers^8,51,52^. In cultures like this, it is important to confirm the UMN identity of eGFP + neurons. We previously reported that the eGFP + neurons located in layer 5 of the motor cortex in UCHL1-eGFP neurons are indeed UMNs^40^. In addition, when UCHL1-eGFP mice were crossed with well-defined ALS models that show progressive UMN loss, not all neurons but these eGFP + neurons declined in numbers^4,40,60^. Even though eGFP expression is present in layer 5 of the brain, it is very specific to UMNs in the motor cortex^40^. Therefore, it is important to perform microdissection, prior to establishing dissociated motor cortex cultures, such that only the motor cortex is included in the study. We thus set up dissociated cultures only from motor cortex so that all eGFP + neurons in culture are UMNs with large pyramidal cell bodies that express Ctip2.

We report that **NU-9** is more effective than riluzole or edaravone, the two FDA-approved drugs for ALS, in improving the health of UMNs diseased due to mSOD1 toxicity, as measured by increased axon outgrowth and neuronal branching/arborization, two important cellular outcome measures for improved UMN health. Most significantly, when **NU-9** is applied together with riluzole or edaravone, there is an additive, but not synergistic, effect.

ALS is a very complex disease, and heterogeneity of patient population hampers drug discovery efforts. In the near future, we will have to build combinatorial drug treatment strategies, including drugs that are tailored specifically for the mechanisms that are perturbed or altered in patients, so that personalized medicine approaches can be developed. This report introduces a novel in vitro platform that utilizes the cellular responses of diseased UMNs as a quantitative outcome measure, reveals that **NU-9** is more effective than riluzole or edaravone, and that combinatorial treatment strategies would have even better outcomes for ALS and other upper motor neurons disease patients.

## Methods

### NU-9

**NU-9** was prepared as described previously^35^.

### Mice

All animal experiments were performed in compliance with the standards set by National Institutes of Health (NIH) and were reviewed and approved by the Northwestern University Institutional Animal Care and Use committee (IACUC approval number # IS00009980). All experiments were conducted in compliance with the ARRIVE guidelines. A 7-day repeat dose toxicity study was performed at the Laboratory Animal House (LAH), Sai Life Science Ltd. (Chrysalis Enclave, Phase II, Hinjewadi, Pune—411 057, Maharashtra, India). **NU-9** was administered to male BALB/c mice once daily for seven consecutive days by oral gavage at a 100 mg/kg/day dosage.

All mice used for cell culture experiments were on C57BL/6 background. Transgenic hemizygous males expressing a high copy number of the human SOD1 gene with a *G93A* mutation (B6SJL-Tg(SOD1*G93A)1Gur/J; The Jackson Laboratory) were bred to hemizygous UCHL1-eGFP females to generate hSOD1^G93^^A^-UeGFP and WT-UeGFP (control) mice. UCHL1-eGFP mice were generated in the Ozdinler Lab; they are reporter lines for UMNs^40^ and are now available at Jackson Laboratory (stock no. 022476). Transgenic mice were identified by PCR amplification of DNA extracted from their tail, as previously described^4,40,41,61^. Mice with different genotypes are abbreviated in the figures as follows: WT-UeGFP (control) mice = G mice; hSOD1^G93A^-UeGFP mice = GS mice.

### Compound preparation and delivery

**NU-9** was prepared as 100 µM stock in dimethylsulfoxide (DMSO) and added to serum free medium [SFM: 0.034 mg/L BSA, 1 mM L-glutamine, 25 U/mL penicillin, 0.025 mg/mL streptomycin, 35 mM glucose, and 0.5% B27 in Neurobasal-A medium (Life Technologies)] at a final concentration of 400 nM (4 µl per 1 ml SFM). Riluzole (Acros Organics) was prepared as a 200 µM stock in DMSO and added to SFM at a final concentration of 500 nM (2.5 µl per 1 ml SFM). Edaravone (Sigma-Aldrich) was prepared as a 1 mM stock in DMSO and added at a final concentration of 1 µM in SFM (1 µl per 1 ml SFM).

### UMN Cultures

Primary motor cortices were isolated from WT-UeGFP or hSOD1^G93A^-UeGFP mice at P3 by microdissection under a fluorescent dissecting scope (Nikon) excluding all neighboring cortical areas such as somatosensory, prefrontal, or cingulate cortex. Isolated motor cortices were dissociated by papain (Worthington Corp., Lakewood, NJ) digestion, and cultured on 18 mm diameter glass coverslips (4 × 10^4^ cells per coverslip, Fisher brand) coated with poly-L-lysine (10 mg/mL, Sigma) as previously described^46^. Neurons were cultured in SFM [0.034 mg/L BSA, 1 mM L-glutamine, 25 U/mL penicillin, 0.025 mg/mL streptomycin, 35 mM glucose, and 0.5% B27 in Neurobasal-A medium (Life Technologies)] in a humidified tissue culture incubator in the presence of 5% CO_2_ at 37 °C. **NU-9** (400 nM), riluzole (500 nM, Acros Organics), and edaravone (1 μM, Sigma-Aldrich) were added at the start of the culture. Cultures were fixed after 3 days in vitro (DIV). These concentrations were chosen based on the concentrations that were calculated and reported to be present in the CNS at their optimum dose, for each compound^10,15,21,24,38^. A total of n = 3 WT-UeGFP (control) mice and n = 3 hSOD1^G93^^A^-UeGFP mice from different litters were used.

### Immunocytochemistry

The antibodies used are as follows: anti-GFP (1:1000, Abcam, Waltham, MA), anti-Ctip2 (1:500, Abcam, Waltham, MA), anti-MAP2 (1:500, Millipore, Temecula, CA), anti-NF-H (1:500, Millipore, Temecula, CA), anti-misfolded SOD1 (1:200, Clone B8H10, MediMabs; Montreal, Quebec, Canada). Briefly, sections were treated with blocking solution (PBS, 0.05% BSA, 2% FBS, 1% Triton X-100, and 0.1% saponin) for 30 min at room temperature and incubated with primary antibody diluted in blocking solution overnight at 4 °C. Secondary fluorescent antibodies (1:500, AlexaFluor-488, -555 or -647 conjugated, Invitrogen) were added to the blocking solution at room temperature for 2 h in the dark. Nuclei were counterstained with DAPI.

### Imaging and quantification

UMNs were quantitatively analyzed for differences in neurite length and arborization complexity. The number of neurons imaged and analyzed for these experiments is as follows: WT-UeGFP SFM, n = 119 neurons from n = 3 mice; hSOD1^G93A^-UeGFP SFM, n = 122 neurons from n = 3 mice; hSOD1^G93A^-UeGFP **NU-9,** n = 115 neurons from n = 3 mice; hSOD1^G93A^-UeGFP riluzole, n = 116 neurons from n = 3 mice; hSOD1^G93A^-UeGFP edaravone, n = 120 neurons from n = 3 mice; hSOD1^G93A^-UeGFP **NU-9** + riluzole, n = 120 neurons from n = 3 mice; hSOD1^G93A^-UeGFP **NU-9** + edaravone, n = 113 neurons from n = 3 mice (Supplemental Table S1). 18 mm coverslips were scanned with an epifluorescent microscope (Nikon) to identify and locate the UMNs based on their GFP expression, and images captured were analyzed using the Neurite Tracer plugin from FIJI (NIH), which enables semi-autonomous tracing to measure the length of the axon. If it was not possible to trace the axon of an individual UMN, for example if they were within a large neuronal/cellular cluster or when UMNs contact each other and it is not possible to discern one from the other, these UMNs were not included in axon measurement and Sholl analyses. All other UMNs that can be identified as a single cell were included in the assay with no additional selection criteria. The longest neurites from UMNs were identified and traced using FIJI (ImageJ, NIH), and the length in micrometers was measured for each neuron. Average neurite length was calculated for each experimental condition for each mouse and reported as the average ± S.E.M. The aggregation of the neurite tracings centered at the soma generates a profile available for Sholl analysis. The number of intersections at 5 µm radius intervals for each neuron was calculated and averaged for each experimental condition for each mouse.

### Statistical analysis

All analyses were performed using Prism software (GraphPad Software). The D'Agostino and Pearson normality test was performed on all datasets. Statistical differences between two groups were determined by unpaired t-test with Welch's correction. Statistical differences between more than two groups were determined by one-way ANOVA followed by the Tukey's post-hoc multiple-comparison test. Two-way ANOVA with Sidak's multiple comparisons test was used to determine the significance of each cell mean with the other cell mean in that row for Sholl analysis. Statistically significant differences were taken at *p* ≤ 0.05.

## Supplementary Information

Supplementary Information 1.

Supplementary Information 2.

Supplementary Information 3.

Supplementary Information 4.
