# Supplementary figures and images for: NU-9 improves health of hSOD1G93A mouse upper motor neurons in vitro, especially in combination with riluzole or edaravone
Source: Sci Rep. 2022 Mar 30;12:5383. doi: 10.1038/s41598-022-09332-4 (PMC8967818; doi:10.1038/s41598-022-09332-4)

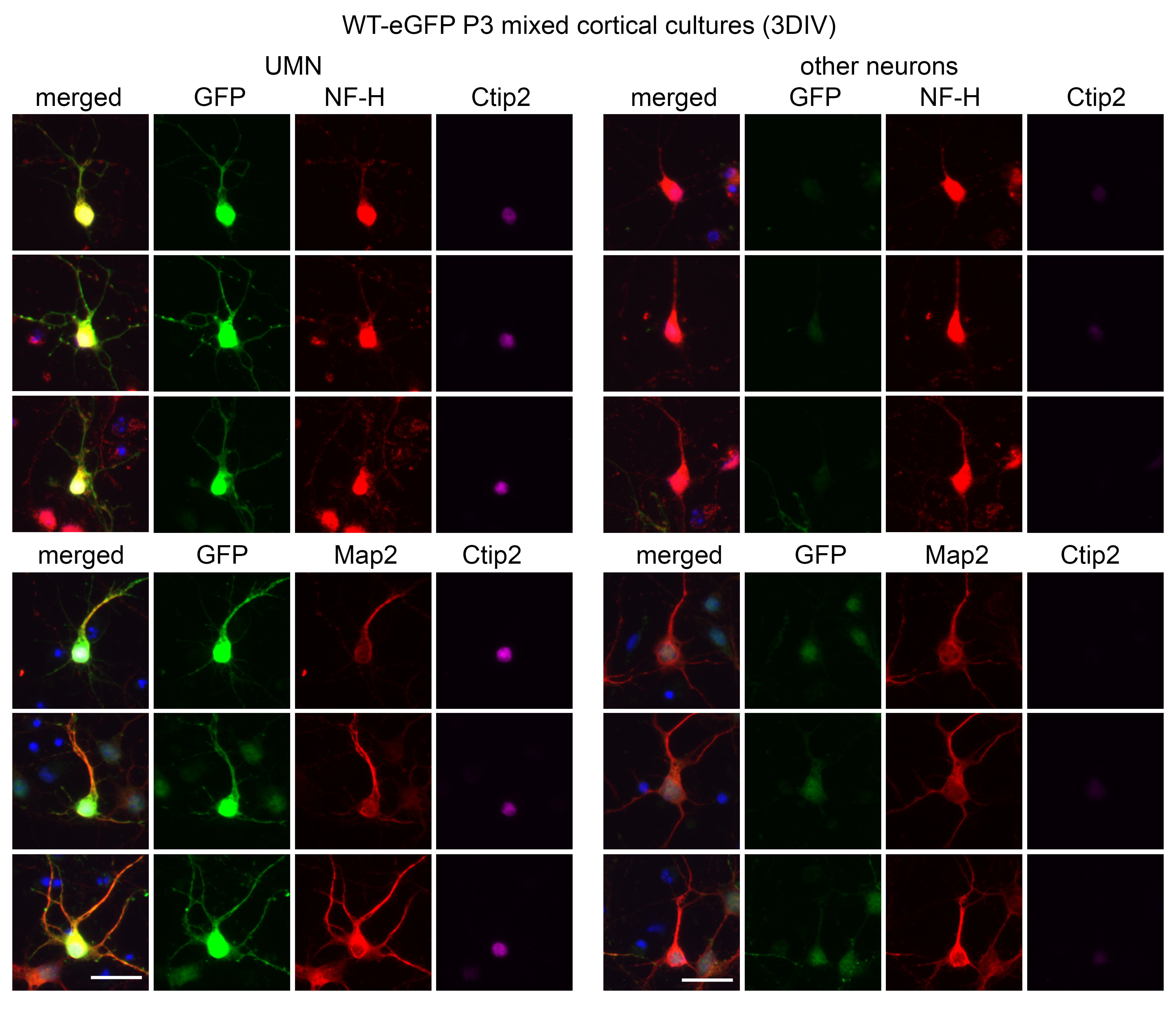

Supplement: Supplementary file 1 — Supplementary Information 1. [file 41598_2022_9332_MOESM1_ESM.tif]
