# Supplementary material for: NU-9 improves health of hSOD1G93A mouse upper motor neurons in vitro, especially in combination with riluzole or edaravone
Source: Sci Rep. 2022 Mar 30;12:5383. doi: 10.1038/s41598-022-09332-4 (PMC8967818; doi:10.1038/s41598-022-09332-4)
Supplement: Supplementary file 4 — Supplementary Information 4. [file 41598_2022_9332_MOESM4_ESM.docx]

**Supplemental Information:**

**Supplementary Fig S1:** **In dissociated cortical cultures isolated from the motor cortex of UCHL1-eGFP mice, UMNs retain their eGFP expression *in vitro*.** Among all other cortical cells (the blue dots (DAPI) represent nuclei of other cells in culture) and neurons (express neuronal markers neurofilament-H (NF-H) and microtubule-associated protein (Map2)), UMNs can be identified based on their eGFP expression and the maintain expression of UMN molecular marker Ctip2. Scale bars = 25 um.

**Supplementary Table S1: Axon length measurements of all neurons included in this study, averages, and statistics.** Length of the longest axon from each neuron is measured, and average values calculated per mouse. Then average ± S.E.M. was calculated for n = 3 mice each per treatment and genotype. For comparison of two samples, unpaired t-test with Welch's correction was used, for comparison of 3 or more samples, one-way ANOVA followed by Tukey's post hoc multiple-comparison test was used to determine statistical significance.

**Supplementary Table S2: Sholl analysis results.** Average number of intersections per radius per mouse with various treatments and pairwise Šídák's multiple comparisons test (Compare each cell mean with the other cell mean in that row).
